# Antibody Exchange: Information extraction of biological antibody donation and a web-portal to find donors and seekers

**DOI:** 10.3390/data2040038

**Published:** 2017-11-21

**Authors:** Sandeep Subramanian, Madhavi K. Ganapathiraju

**Affiliations:** 1 Language Technologies Institute, Carnegie Mellon University; 2 Department of Biomedical Informatics, and Intelligent Systems Program, University of Pittsburgh

**Keywords:** data exchange, resource donations, text mining

## Abstract

Bio-molecular reagents like antibodies required in experimental biology are expensive and their effectiveness, among other things, is critical to the success of the experiment. Although such resources are sometimes donated by one investigator to another through personal communication between the two, there is no previous study to our knowledge on the extent of such donations, nor a central platform that directs resource seekers to donors. In this paper, we describe, to our knowledge, a first attempt at building a web-portal titled Antibody Exchange (or more general ‘Bio-Resource Exchange’) that attempts to bridge this gap between resource seekers and donors in the domain of experimental biology. Users on this portal can request for or donate antibodies, cell-lines and DNA Constructs. This resource could also serve as a crowd-sourced database of resources for experimental biology. Further, we also studied the extent of antibody donations by mining the acknowledgement sections of scientific articles. Specifically, we extracted the name of the donor, his/her affiliation and the name of the antibody for every donation by parsing the acknowledgements sections of articles. To extract annotations at this level, we adopted two approaches – a rule based algorithm and a bootstrapped pattern learning algorithm. The algorithms extracted donor names, affiliations and antibody names with average accuracies of 57% and 62% respectively. We also created a dataset of 50 expert-annotated acknowledgements sections that will serve as a gold standard dataset to evaluate extraction algorithms in the future.

## 1. Introduction

Antibodies and other such wet-lab reagents are vital resources in a variety of experiments in molecular biology. These resources are expensive and their quality is crucial for the success of the experiment. For those conducting these experiments, it would be extremely valuable when these reagents become available in spare quantities in one lab, they are then donated to others when required. This donation will be even more useful if the donor lab has already tested the quality of the resource. For example, a research group that studies the protein HMGB1 extensively, might have a reliable and well-tested antibody for it, and could potentially donate some of it to colleagues or collaborators who may need it. Such donations, where possible, could help unfunded junior investigators to carry out experiments that they otherwise could not afford. Further, such acts of generosity could spark collaborations between research groups and serve as a means to connect researchers with similar expertise.

Even in the strongly connected world that we are in today, researchers, unaware of a group that may have the reagents in close proximity within their organization, typically turn to vendors whose information is readily available online. Increasingly, there has been a trend towards open resource sharing. For example, open source software, open data sharing and open access of manuscript publishing have become pervasive and have accelerated advancement of science.

In these open sharing environments, what are the factors that drive people to do social good? While some individuals have altruistic motives such as contributing to the advancement of science and encouraging junior investigators, there are others who build a reputation for being highly visible donors or build goodwill for future reciprocations. How feasible is it to share material resources among research groups, given that they cannot be shared simply over the Internet?

In this work, we studied the extent to which researchers share biological reagents, specifically antibodies, by parsing the acknowledgements sections of papers available in PubMed Central. Encouraged by what we found, we developed a web portal to connect donors with seekers of reagents to facilitate and promote sharing of such resources. This portal can serve as a means for people to find locally available resources for their experiments.

The amount of bio-medically relevant content is increasing at an unprecedented rate; two new articles are published on PubMed every minute [1]. Therefore, information extraction from text documents has seen several advancements over the past decade [2–4]. The BioCreative and BioNLP workshop initiatives were created to evaluate text mining and information extraction approaches. Tasks ranging from named entity recognition (NER) on genes, drugs and chemical compounds to protein-protein interaction extraction from PubMed have been a part of these initiatives [5, 6]. Further, GENIA [7] is a dataset pertinent to text mining of bio literature and has played an important role in the advancement of Biomedical Natural Language Processing.

Riloff and Jones [8] pioneered an information extraction algorithm that iteratively learns rules for extracting relevant information and in turn uses the information to learn new rules. This approach to learning is often referred to as bootstrapping and is in practice to-date [9–11]. Some of the biggest and most successful information extraction systems like Never Ending Language Learner (NELL) [12] have used bootstrapping effectively even in the biomedical domain [13]. We adopt this as one of our methods to extract information from literature. The NLP research community has largely stuck to machine learning approaches for information extraction until very recently when rule based systems have seen some resurfacing, while the industry has always stuck to the latter [14]. Rule based information extraction systems have the advantage of being interpretable and can be fine-tuned easily [11]. In this work, we experiment with using a purely hand-engineered rule based extraction system and compare its performance with bootstrapped pattern learning system. Recently, bio-literature has been mined to index and curate bioinformatics and biomedical resources [15–17]. Several examples are presented in our prior work [18]. Duck et al [19] present a literature mining approach to quantifying the use of resource in *computational biology* while de la Calle et al [17] do the same for resources in *bioinformatics*. In contrast, we focus on wet-lab resources. Ozyurt et al [15] develop a holistic resource that has software, databases and tissue banks but does not contain antibodies or attempt to understand the phenomenon of bio resource donation in literature.

## 2. Materials and Methods

Researchers acknowledge donations from others by thanking them in the acknowledgements section of their published work. In this particular work, we focused on studying acknowledgements pertaining to antibody donations. We mined full-text articles from PubMed Central to extract information at coarse and fine granularities. At the coarse level, we extracted the entire acknowledgement section if a case-insensitive search on the entire acknowledgement section contains the word “antibody” or “antibodies”. Authors however, tend to acknowledge multiple things in this section such as manuscript reading, instrument usage and their funding sources. For example, the acknowledgement “We thank Peter Merrifield and Stefano Schiaffino for providing antibodies. This work was supported by grants from the Medical Research Council of Canada. K.E.M.H is a Killam Scholar of the Montreal Neurological Institute.” contains information extraneous to the task we are focused on. We therefore had to develop extraction algorithms that can carefully extract out donor names, donor affiliations and antibody names from entire acknowledgement sections.

### *A.* Data Acquisition

PubMed Central provides full-text access to all of its open access papers https://www.ncbi.nlm.nih.gov/pmc/. As of April 2015, according to our statistics it consisted of a total of 1,000,148 open access papers. These papers are available for download, free of cost and formatted in XML. We parsed these to extract the acknowledgements section of every paper and searched for a reference to an antibody donation within it. Since generating the entire XML parse tree of every paper was computationally expensive, we ran a shallow parse using regular expressions to parse out just the acknowledgements section.

A crude extraction approach for this task was done using a case insensitive search for the word “antibody” and “antibodies” in the acknowledgements section, which returned 6,533 instances across all papers in all journals. Only a very small fraction of these did not contain a reference to an antibody donation. For example, the sentence “We’d like to thank Doris Thelian for her expert advice on antibody cocktails and flow cytometry data analysis” has absolutely no reference to donation of an antibody.

We then analyzed the extracted paragraphs using information extraction algorithms that we will describe in detail in the subsequent sections to determine the antibody donor name, affiliation and the name of the antibody.

### *B.* Rule Based Extraction

Rule based systems can easily exploit the formal and consistent nature of writing in the acknowledgements section of scientific articles. Rule based information extraction systems that search key-word context windows have been employed with success in the past. The context in which a word occurs (i.e.) the words surrounding it, has been studied extensively [20–23]. We formulate heuristically determined search rules for information extraction within these word contexts.

**Fig. 1:**
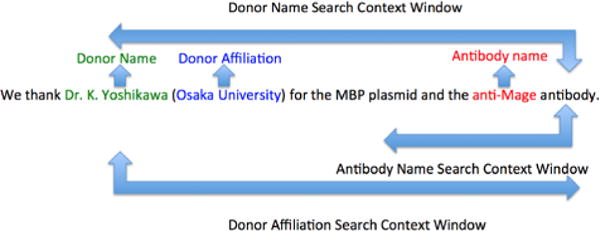
Rule based extraction

We observe that the antibody names were mostly written in ‘TARGET^1^ antibody” form (Fig. 1), but also occasionally as “antibody of/to TARGET”; for example: “Dr. Y. Nishiyama is thanked for the antibody to UL7” is written in passive voice, which would require searching the right context instead. We assert that the first word within the left context window of the word “antibody” that is not in the English dictionary or a named entity, is the name of the antibody that was donated. If no such word is found in the left context, we then proceed to search the right-context. The size of the left and right context windows is set at 4 words, determined after examining the paragraphs from the high-level extraction step. Further, we also search the left context window for any tags (primary, secondary, monoclonal, polyclonal) that may associated with the antibody. While an NER system for antibodies would have been ideal, biomedical NER systems such as BANNER [24] are incapable of tagging antibodies nor is there a corpus from which a supervised one can be trained.

While extracting the name of the donor, we do not fix the size of the context window in which we search. Instead, we keep searching the left context of the antibody name until a named entity labeled as a person is encountered. We found that the name of a donor is typically located far away from the antibody. We used MIT’s Information Extraction Library^2^ (MITIE) for NER that identifies named entities and provides labels for them like ‘person’, ‘organization’ and ‘location’. Another observation about the nature of acknowledgements in biomedical literature was that a person’s affiliation almost always occurred immediately after his/her name within brackets. We used this to label the donor’s affiliation as the closest organization extracted by our NER in his/her right context while still being on the left context of the word “antibody” or “antibodies”.

### *C.* Bootstrapped pattern learning

While rule-based extraction systems are capable of extracting entities with high precision, they require rules to be explicitly defined. This also prevents them from being easily adapted to new domains. Bootstrapping alleviates this problem by automatically learning phrases/patterns that identify entities of interest from seeded ground-truth annotations. The following subsection describes the bootstrapping algorithm that we used to learn extraction rules.

**Fig. 2:**
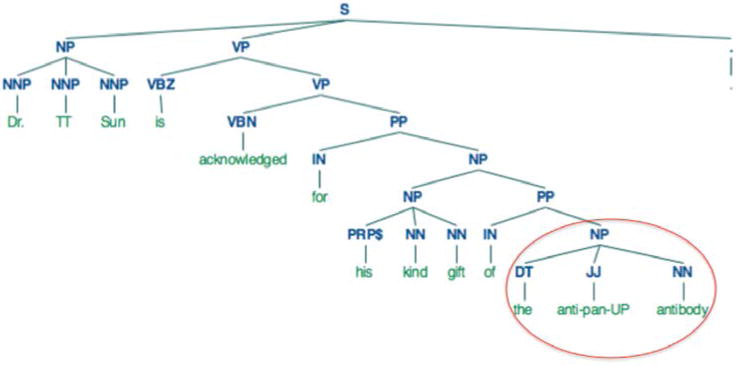
Constituency parse of a sentence to find an extraction rule

**Fig. 3:**
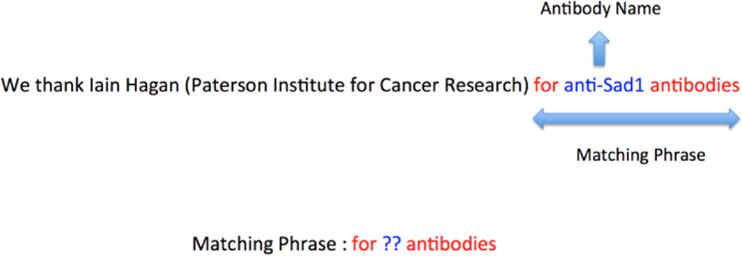
Example extraction rule

We used bootstrapping to identify antibody names only, using the context-based approach as described in the Rule Based Extraction to identify the donor names, affiliations and antibody names. The algorithm is as follows:

- Seed an initial set of antibody names.
- For every sentence that contains any of the seeded antibody names, run a constituency parse to extract the leaves of immediate parent noun phrase as shown in Fig. 2 and replace the seeded antibody with a wildcard ‘TARGET’.
- These phrases constitute the learned patterns. (Fig. 3)
- Extract new antibody names using these learned patterns by pattern matching any of these patterns with every sentence.
- Repeat antibody-name extraction and rule-learning steps iteratively.

Bootstrapping algorithms learn new patterns and ground-truth iteratively [2][25, 26]. The algorithm constitutes extracting antibody names either from the initial seed or from the patterns learned thus far, and then updating patterns from the current set of antibody names extracted. We observed that best performance was achieved after 2 iterations, whereas more iterations introduced noisy extraction rules.

Some of the patterns learned by this algorithm starting with 40 antibody names as seeds are:

- the mouse TARGET antibody
- rabbit TARGET antibodies
- TARGET monoclonal antibodies
- TARGET antibody
- antibody to TARGET

### *D.* Human Annotations

There is no dataset with ground-truth annotations for evaluating these algorithms. So we undertook collection of human annotations for 50 randomly sampled acknowledgement sections. Biologists familiar with this domain were asked to manually annotate donor names, his/her affiliation, the name of the antibody and any of its attributes. We also asked the annotators to identify other bio-resources (e.g. cell-lines) that they could find in the acknowledgements and annotate them with labels describing the resource and the resource name for future work along this line. Further, we also asked them to annotate people or organizations in the acknowledgements that were not part of a donation of a bio-resource for potential application in NER tasks.

**Fig. 4:**
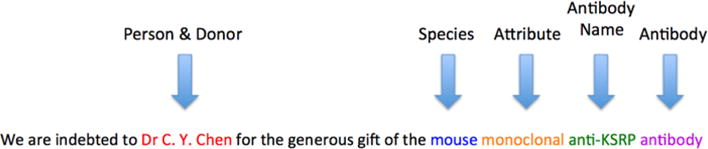
Example annotation of an antibody donation

**Fig. 5:**
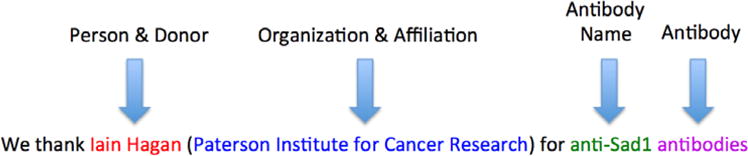
Example annotation of an antibody donation

**Fig. 6:**
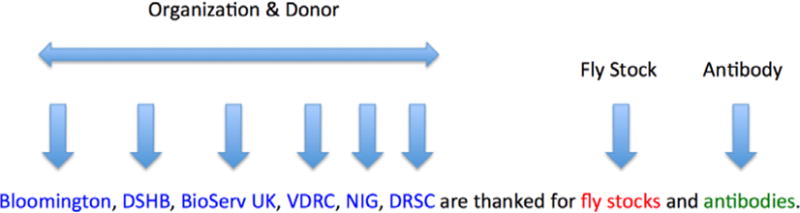
Example annotation of a fly stock donation

Example annotations of sentences describing only antibody donations are shown in Fig. 4 and Fig. 5 and annotations of sentences containing other bio-resources are shown in Fig. 6.

**Fig. 7:**
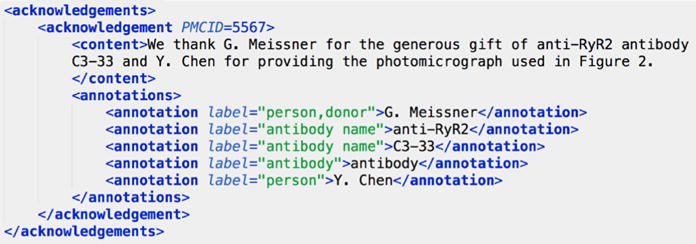
Human annotations formatted in XML

Five biologists participated in the annotation process. Fifty abstracts were annotated overall, of which 18 were annotated by at least 2 individuals. Inter-annotator agreement was 75%. We foresee these annotations being used as ground-truth for other researchers to evaluate their algorithms on the same task. More generally, these annotations could be used to train information extraction and named entity recognition systems. The annotations are formatted in XML, a snippet of which can be seen in Fig. 7.

The dataset of annotated acknowledgements is provided in Supplementary File 1.

## 3. Results and Discussion

We studied frequent donors (people & organizations), frequently donated antibodies, and donation trends across journals, and trends over time. These results are presented for both approaches.

### *A.* Rule Based Approach

The rule-based approach extracted a total of 7,589 antibody donations. The number of extracted donations exceeds the number of acknowledgement sections because the algorithm is capable of extracting multiple donations within the same acknowledgement section. Table 1 contains the top 5 donor names irrespective of their affiliation.

Table 2 contains the top 5 donor-affiliation pairs.

Our approach suffers from some weaknesses – the NER system tagged “Albert Einstein College of Medicine” as a person. Also, it is incapable of identifying different ways of writing a donor name (Keith Gull vs K. Gull vs Gull, K) or affiliation (University of Oxford vs Oxford University).

Table 3 contains the organizations that donated the most antibodies.

Table 4 contains the most frequently donated antibodies.

Table 5 contains the journals that had the most references to an antibody donation. Note that these journals are completely open access, however all their articles are available in the data we processed.

**Fig. 8:**
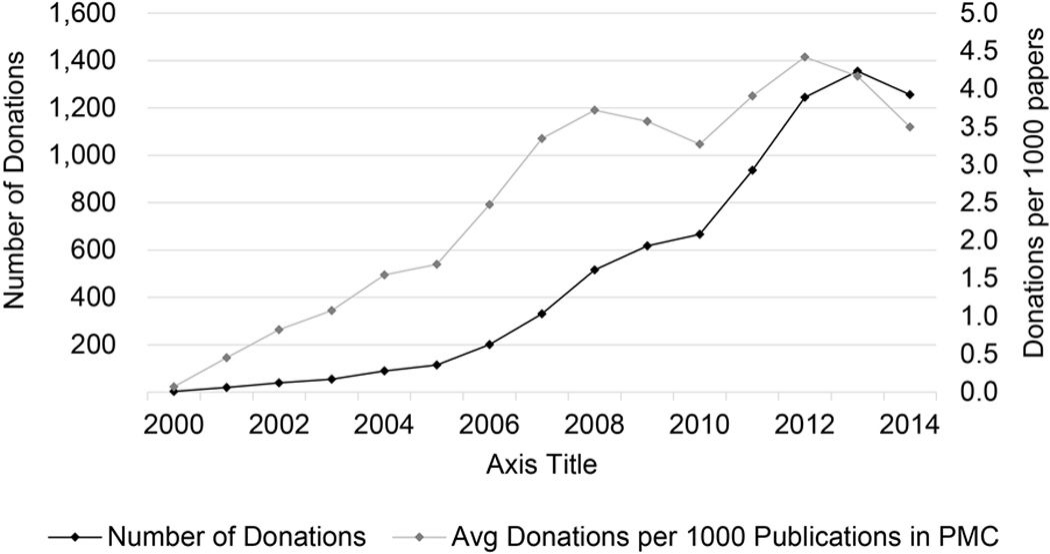
Year vs number of donations extracted in that year

Fig. 8 shows a plot of the number of antibody donations extracted papers published during the years 2000 to 2014. The data the nature of open-access publications and their deposition in PubMed Central have seen increasing adoption during this period because of which the number of publications per year grew in PMC, from around 40 thousand in 2000 to about 400,000 thousand in 2014. To normalize for this effect, we also show ‘donations per 1000 PMC articles’, with a secondary axis on the right-side of the figure. We counted multiple donations mentioned in the same paper (of different antibodies or by different donors) as distinct entities; however, these are rare occasions and would not significantly alter normalized values. We can see that the donations themselves, or the practice of acknowledgement in the manuscripts, have increased over the years 2000-2014.

### *B.* Bootstrapped Pattern Learning

The bootstrapped pattern learning algorithm extracted a total of 7,864 antibody donations. Table 6 contains the top 5 donors independent of their affiliation.

Table 7 contains the top 5 donor-affiliation pairs.

Table 8 contains the organizations that donated the most antibodies.

Table 9 below contains the antibody names that were donated the most frequently.

Table 10 contains the journals that had the most references to an antibody donation.

The plot showing the temporal donation trend for this approach was identical to the previous approach and is therefore not included in this section.

## 4. Extraction Evaluation

### *A.* Evaluation

We evaluated the performance of our algorithms, by comparing them to any of the human labeled annotations. We report the accuracies in Table 11. It is evident that we are able to extract characteristics about an antibody using both our proposed approaches. The bootstrapped pattern learning algorithm achieves better performance than the simple rule-based approach at extracting donor and antibody names but doesn’t do as well with extracting affiliations.

## 5. Bio-Resource Exchange Web Portal

**Fig. 9:**
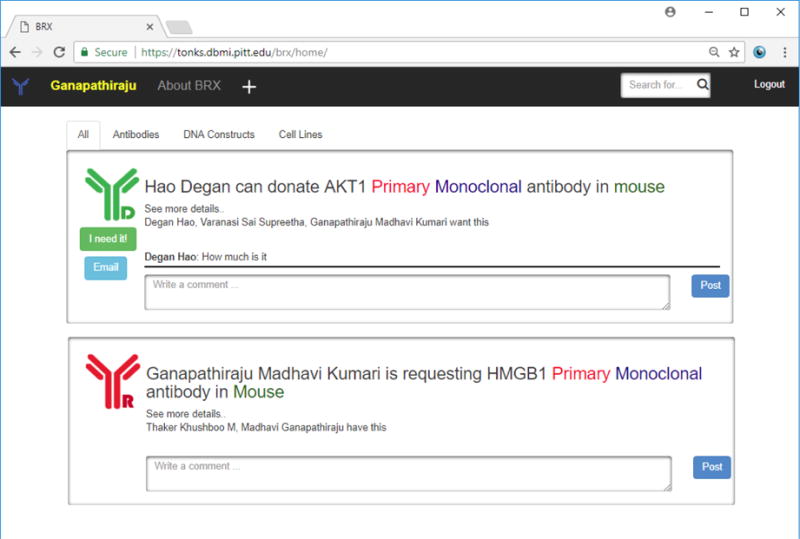
Screenshot of the Web-Portal

We developed a resource-sharing web-portal called Bio-Resource Exchange (BRX) available at http://tonks.dbmi.pitt.edu/brx. It is built modularly, with the ability to be a generic resource-sharing platform. It allows users to make requests for or donate resources via a simple customizable form for each resource. At present, resources on BRX include antibodies, DNA constructs and cell-lines. The moment a form is filled in by a user, it appears on a bulletin board (analogous to a news feed on social networking websites) that is visible to all other users in the system (Fig. 9). A user’s news feed may be filtered based on the type of research he/she is looking for. It allows users to search for specific information, for example, particular antibodies or cell-lines, or posts by specific individuals. BRX also allows users to comment on posts and also puts them in touch with the author via email.

BRX was developed using the Django web framework with a MySQL backend database. Separate tables were created for each resource type to allow each of them to have different attributes using Django’s ORM (Object-relation Mapping). Foreign keys to this table were made to store comments and email correspondences. The rest of the backend elements are designed to make the addition of a new resource extremely simple. Third-party authentication elements on BRX were built using an open source Oauth2 library^3^. For University of Pittsburgh users exclusively, we used LDAP (Lightweight Directory Access Protocol) authentication to let users sign in with their university email accounts. Front-end elements were built using twitter-bootstrap, Vanilla JavaScript and jQuery. In the future, the front-end could include leaderboards of universities, organizations and people who have donated the most resources to promote healthy competition.

The results from mining literature haven’t been incorporated into the website, because the posts are tied to individual profiles, i.e. of donors and seekers. Unless a user registers and posts the information that they are seeking/donating antibodies or other resources, it does not appear on the web portal.

## 6. Conclusions

We carried out text-mining over acknowledgement sections of open-access articles to study the extent of antibody donations reported so far. We first created a dataset of 50 expert-annotated acknowledgements sections that will be useful for algorithm development and evaluation purposes in studying such donations. Using NLP techniques, we extracted the name of the donor, his/her affiliation and the name of the antibody for every donation by parsing the sentences by adopting two approaches – a rule based algorithm and a bootstrapped pattern learning algorithm, and achieved average accuracies of 57% and 62% respectively.

We also developed a web-portal, Bio-Resource Exchange that attempts to connect biologists seeking antibodies, cell-lines or DNA-constructs to potential donors. We expect that it would bridge a gap between resource seekers and donors in the domain of experimental biology. Users on this portal can post information that they are either seeking a specific antibody, cell-line or DNA construct, or that they are in a position to donate them. It allows other registered users to comment on such posts. Thus, this resource could grow into a crowd-sourced database of resources for experimental biology.

## 7. Limitations

A significant limitation of this work is that the text-mining methods adopted were extremely simple and involved the use of several heuristics owing to limited data and the absence of a labeled corpus for such a task. There exist no corpora from which named entity taggers can be learned to recognized antibody names in a supervised manner and therefore more contemporary methods such as CRFs [27] or neural methods [28] could not be adopted. While integrating results from text-mining is trivial from an implementation perspective, some thought has to be put into curating the data that goes onto the web page. For example, attributing an antibody with an incorrect donor could lead to problems when contacting him/her. Further, we found it difficult to get biologists to annotate a large collection of our dataset and so had to distribute our data across many of them thereby inducing a minimal amount of noise in the annotations. Soft matching constraints when evaluating our models could provide deeper insights into the model’s strengths and weaknesses. At present, the XML dataset does not contain the character offsets of each annotation within the paragraph – this is an extension we foresee adding in future releases of this dataset.

## Supplementary Material

Supplemental file

## Figures and Tables

**Table 1:** Top donors by name

Donor	Number of Donations
Keith Gull	32
Albert Einstein College of Medicine	15
Peter Davies	12
K. Gull	10
Hugo Bellen	10

**Table 2:** Top donors by donor-affiliation pairs

Donor	Affiliation	Number of Donations
Keith Gull	University of Oxford	6
Keith Gull	Oxford University	5
Gary Ward	University of Vermont	4
K. Mackie	Indiana University	3
Yoshihiko Funae	Oosaka City University	3

**Table 3:** Top donors by organization

Donor	Number of Donations
University of California	24
NIH	19
Rockefeller University	15
Harvard Medical School	15
University of Pennsylvania	12

**Table 4:** Most frequently donated antibodies

Antibody Name	Number of Donations
plasmids	111
autoantibody	31
DSHB	28
anti-tubulin	14
anti-GFP	14

**Table 5:** Journals with the most donations

Journal	Number of Donations
PLoS One	2,894
PLoS Genetics	667
PLoS Pathology	536
PLoS ONE	294
PLoS Biology	286

**Table 6:** Top donors by name

Donor	Number of Donations
Keith Gull	24
Albert Einstein College of Medicine	20
Erich Buchner	11
Charles Rice	11
K. Gull	10

**Table 7:** Top donors by donor-affiliation pair

Donor	Affiliation	Number of Donations
Dr. Charles Rice	Rockefeller University	9
Steven S. Gross	Weill Medical College	8
Harold Gainer	NIH	7
Keith Gull	University of Oxford	7
Gary Ward	University of Vermont	6

**Table 8:** Top donors by organization

Donor	Number of Donations
NIH	23
Harvard Medical School	22
Rockefeller University	21
University of California	20
University of Pennsylvania	20

**Table 9:** Most frequently donated antibodies

Antibody Name	Number of Donations
plasmids	74
anti-mouse	30
anti-gfp	18
anti-tubulin	12
anti-actin	10

**Table 10:** Most frequently donated antibodies

Journal	Number of Donations
PLoS One	3174
PLoS Pathology	671
PLoS Genetics	577
PLoS Biology	306
PLoS ONE	301

**Table 11:** Extraction results

		Accuracy		
Approach				
	Donor	Affiliation	Antibody Name	Mean
Rule Based	50%	70%	50%	57%
Bootstrapped Pattern Learning	57%	66%	64%	62%

## Footnotes

1 TARGET is the name of a protein or other biomolecule that the antibody binds to

2 https://github.com/mit-nlp/MITIE

3 https://github.com/omab/python-social-auth

## References

[1] Nawaz R, Thompson P, Ananiadou S (2013). Negated bio-events: analysis and identification. BMC bioinformatics.

[2] Finkel JR, Grenager T, Manning C (2005). Incorporating non-local information into information extraction
systems by gibbs sampling. Proceedings of the 43rd Annual Meeting on Association for Computational
Linguistics.

[3] Banko M (2007). Open information extraction for the web. IJCAI.

[4] Soderland S (1999). Learning information extraction rules for semi-structured and
free text. Machine learning.

[5] Hirschman L (2005). Overview of BioCreAtIvE: critical assessment of information
extraction for biology. BMC bioinformatics.

[6] Kim JD (2011). Overview of BioNLP shared task 2011. Proceedings of the BioNLP Shared Task 2011 Workshop.

[7] Kim JD (2003). GENIA corpus—a semantically annotated corpus for
bio-textmining. Bioinformatics.

[8] Riloff E, Jones R (1999). Learning dictionaries for information extraction by multi-level
bootstrapping. AAAI/IAAI.

[9] Dempster AP, Laird NM, Rubin DB (1977). Maximum likelihood from incomplete data via the EM
algorithm. Journal of the royal statistical society Series B
(methodological).

[10] Gupta S, Manning CD (2014). Spied: Stanford pattern-based information extraction and
diagnostics. Sponsor: Idibon.

[11] Gupta S, Manning CD (2014). Improved Pattern Learning for Bootstrapped Entity
Extraction. CoNLL-2014.

[12] Carlson A (2010). Toward an Architecture for Never-Ending Language
Learning. AAAI.

[13] Movshovitz-Attias D, Cohen WW (2012). Bootstrapping biomedical ontologies for scientific text using
nell. Proceedings of the 2012 Workshop on Biomedical Natural Language
Processing.

[14] Chiticariu L, Li Y, Reiss FR (2013). Rule-Based Information Extraction is Dead! Long Live
Rule-Based Information Extraction Systems!. EMNLP.

[15] Ozyurt IB (2016). Resource Disambiguator for the Web: Extracting Biomedical
Resources and Their Citations from the Scientific Literature. PLoS One.

[16] Petersen R, Kempler G, Barklis E (1991). A stem cell-specific silencer in the primer-binding site of a
retrovirus. Mol Cell Biol.

[17] de la Calle G (2009). BIRI: a new approach for automatically discovering and indexing
available public bioinformatics resources from the
literature. BMC Bioinformatics.

[18] Roth A, Subramanian S, Ganapathiraju MK (2015). Towards extracting supporting information about predicted
protein-protein interactions. IEEE/ACM Trans Comput Biol Bioinform.

[19] Duck G (2013). bioNerDS: exploring bioinformatics’ database and software
use through literature mining. BMC bioinformatics.

[20] Mikolov T (2013). Distributed representations of words and phrases and their
compositionality. Advances in neural information processing systems.

[21] Pennington J, Socher R, Manning CD (2014). Glove: Global vectors for word representation. Proceedings of the Empiricial Methods in Natural Language Processing
(EMNLP 2014).

[22] Turian J, Ratinov L, Bengio Y (2010). Word representations: a simple and general method for
semi-supervised learning. Proceedings of the 48th annual meeting of the association for
computational linguistics.

[23] Tang B (2014). Evaluating word representation features in biomedical named
entity recognition tasks. Biomed Res Int.

[24] Leaman R, Gonzalez G (2008). BANNER: an executable survey of advances in biomedical named
entity recognition. Pacific symposium on biocomputing.

[25] Wu X, Fan J, Subramanian KR (2002). B-EM: A classifier incorporating bootstrap with em approach for
data mining. Proceedings of the eighth ACM SIGKDD international conference on
Knowledge discovery and data mining.

[26] McCallumzy A, Nigamy K (1999). Text classification by bootstrapping with keywords, EM and
shrinkage.

[27] Settles B (2004). Biomedical named entity recognition using conditional random
fields and rich feature sets. Proceedings of the International Joint Workshop on Natural Language
Processing in Biomedicine and its Applications.

[28] Lample G (2016). Neural architectures for named entity recognition. arXiv preprint arXiv:1603.01360.

